# Mesenchymal Stromal Cells from Fetal and Maternal Placenta Possess Key Similarities and Differences: Potential Implications for Their Applications in Regenerative Medicine

**DOI:** 10.3390/cells9010127

**Published:** 2020-01-06

**Authors:** Andrea Papait, Elsa Vertua, Marta Magatti, Sabrina Ceccariglia, Silvia De Munari, Antonietta Rosa Silini, Michal Sheleg, Racheli Ofir, Ornella Parolini

**Affiliations:** 1 Centro di Ricerca E. Menni, Fondazione Poliambulanza, 25124 Brescia, Italy; andrea.papait@poliambulanza.it (A.P.); elsa.vertua@poliambulanza.it (E.V.); marta.magatti@poliambulanza.it (M.M.); silvia.demunari@poliambulanza.it (S.D.M.); antonietta.silini@poliambulanza.it (A.R.S.); 2 Department of Life Science and Public Health, Università Cattolica del Sacro Cuore, 00168 Rome, Italy; sabrina.ceccariglia@unicatt.it; 3 Fondazione Policlinico Universitario “Agostino Gemelli” IRCCS, Largo A. Gemelli, 8, 00168 Rome, Italy; 4 Pluristem LTD, Haifa 31905, Israel; michals@Pluristem.com (M.S.); racheli@pluristem.com (R.O.)

**Keywords:** human placenta, amniotic membrane, immunomodulation, mesenchymal stromal cells, PLX: PLacenta expanded mesenchymal-like adherent stromal cells

## Abstract

Placenta-derived mesenchymal stromal cells (MSC) have attracted more attention for their immune modulatory properties and poor immunogenicity, which makes them suitable for allogeneic transplantation. Although MSC isolated from different areas of the placenta share several features, they also present significant biological differences, which might point to distinct clinical applications. Hence, we compared cells from full term placenta distinguishing them on the basis of their origin, either maternal or fetal. We used cells developed by Pluristem LTD: PLacenta expanded mesenchymal-like adherent stromal cells (PLX), maternal-derived cells (PLX-PAD), fetal-derived cells (PLX-R18), and amniotic membrane-derived MSC (hAMSC). We compared immune modulatory properties evaluating effects on T-lymphocyte proliferation, expression of cytotoxicity markers, T-helper and T-regulatory cell polarization, and monocyte differentiation toward antigen presenting cells (APC). Furthermore, we investigated cell immunogenicity. We show that MSCs and MSC-like cells from both fetal and maternal sources present immune modulatory properties versus lymphoid (T cells) and myeloid (APC) cells, whereby fetal-derived cells (PLX-R18 and hAMSC) have a stronger capacity to modulate immune cell proliferation and differentiation. Our results emphasize the importance of understanding the cell origin and characteristics in order to obtain a desired result, such as modulation of the inflammatory response that is critical in fostering regenerative processes.

## 1. Introduction

In the past several decades, mesenchymal stromal cells (MSC) have been the subject of extensive studies, representing a hypothetical magic bullet in regenerative medicine. Initially, MSC attracted much attention due to their low immunogenicity and differentiation capability. However, currently, they are widely recognized for their immunomodulatory properties. Over the years, it has been possible to identify MSC in different tissues and, over time, it has been appreciated that different tissues harbor MSC with peculiar characteristics/properties. More specifically, in the past two decades, the human placenta has become a consolidated source of MSC that possess unique properties.

Placenta plays an essential role in supporting the development of the fetus and represents an important reservoir of transient progenitor and stem cells. To date, MSC have been isolated from different regions of the placenta of both fetal and maternal origin.

According to the First International Workshop on Placenta-Derived Stem Cells held in Brescia, Italy in 2007 [1], four major regions of fetal placenta are identified in which each harbor potential stem/progenitor cells identified as: human amniotic epithelial cells (hAECs), human amniotic mesenchymal stromal cells (hAMSCs), human chorionic mesenchymal stromal cells (hCMSCs), and human chorionic trophoblastic cells (hCTCs) [1,2,3,4,5]. Mesenchymal stromal/stem cells (MSCs) have also been isolated from other placental tissues, such as the chorionic villi [6,7,8,9,10,11], the maternal decidua basalis [4,12,13], and from different compartments of the umbilical cord, such as the Wharton’s jelly [14,15,16].

The maternal component of the placenta, which is in direct contact with extra-embryonic fetal tissues, the decidua, has been the subject of intensive investigation in an attempt to explain the mechanisms involved in the delicate immunological balance that governs pregnancy. Within the decidua, different immune cells can be identified, whether they are T lymphocytes [17,18,19], macrophages [20], or natural killer (NK) cells [21,22], with proportions that change during pregnancy, play a significant role in regulating the implantation, placentation, and maintenance of pregnancy, and, ultimately, impact the maternal immune system [21,23,24]. The unique immunological setting between mothers and the fetus during pregnancy has led to the hypothesis that the placenta fosters cells with immunological properties being critical in maintaining feto-maternal tolerance during pregnancy.

In line with this hypothesis, MSC isolated from different placenta regions have been shown to possess immune modulatory properties [25,26,27,28,29]. In addition, the regions from which placental cells are taken has been shown to impact properties such as differentiation, angiogenesis, and ability to inhibit T-lymphocyte proliferation [30,31,32,33], which potentially has a significant impact on their applications in regenerative medicine as well as in the treatment of inflammatory and autoimmune disorders.

A current open and critical aspect for the applications of MSC in regenerative medicine is the origin of tissue and inherent heterogeneity that it poses. Fetal membranes have been shown to possess areas with different structural characteristics [34] and mitochondrial activity [35]. Moreover, the maternal-fetal immune interactions during gestation could influence the immunomodulatory properties of MSC isolated from maternal and fetal tissues, and some differences have been reported in the ability to impact proliferation [32,33]. However, these studies use a non-specific stimulus such as phytohaemagglutinin (PHA), which has little relevance to the in vivo setting. Thus, given that MSC isolated from specific placenta regions (e.g. amniotic membrane, umbilical cord, chorionic villi) have been shown to possess immune modulatory properties, and given that other properties (such as structural characteristics and mitochondrial activity) have been shown to differ based on the specific region of placenta from which the MSC were isolated, we hypothesized that the maternal-fetal immune interactions during gestation could influence the immunomodulatory properties of MSC isolated from maternal and fetal tissues. In this study, we performed a detailed comparison of the immunological properties of MSC isolated from maternal and fetal components of human term placenta. In addition, we use both research and standardized cell preparations and good manufacturing practice (GMP) products, which bypasses heterogeneity associated with differing cell isolation and culture conditions that could, in turn, influence their immunological properties.

We compared maternal MSC-like cells (PLX-PAD) and two different fetal-derived cells known as PLX-R18 [36,37,38,39,40] and a well-characterized MSC population from the amniotic membrane [41]. Placental expanded (PLX) are of a good manufacturing practice (GMP)-grade clinical investigational product that are prepared using a 3-dimensional (3D) bioreactor-based cell growth platform. The two products are currently being investigated for the treatment of muscle injury following hip fracture and critical limb ischemia (CLI) for PLX-PAD and bone marrow recovery following incomplete hematopoietic cell transplantation (HCT) for PLX-R18 as reported in the ClinicalTrials.gov website, available online: https://clinicaltrials.gov/ (accessed on 04/01/2020). Hence, we analyzed and compared the capacity of maternal and fetal MSC to (a) inhibit T lymphocyte proliferation, (b) modulate the expression of cytotoxicity markers upon activation with CD3/CD28 mAbs, (c) impact the Th subset and Treg polarization, and (d) affect monocyte differentiation toward antigen presenting cells [M1 macrophages and mature dendritic cells (mDC)] and skew toward the M2 phenotype. Furthermore, we investigated the immunogenicity of PLX-PAD cells evaluated as the capacity of these cells to spontaneously induce lymphocyte proliferation in the absence of specific stimuli.

Our findings demonstrate that placenta-derived cells, and, most of all, fetal-derived cells directly impact the immune response by interfering with T-cell activation and by modulating antigen presenting cells (APC) differentiation. These results underline the importance of the origin of cells and, for the first time, provide a vast characterization of two GMP products that approved clinical investigational products. Our observations could, therefore, be useful in guiding clinical decisions as to which placenta-derived cell population could potentially be more promising/apt for specific immunological disorders.

## 2. Materials and Methods

### 2.1. Ethics Statements

For the amniotic membrane-derived MSC (hAMSC), human term placentae (*n* = 11) were collected from healthy women after vaginal delivery or caesarean section at term after obtaining informed written consent, according to the guidelines set by the local ethical committee “Comitato Etico Provinciale di Brescia”, Italy (number NP 2243, 19 January 2016).

PLX cells are collected from healthy women undergoing an elective caesarean section. The placenta donors sign an informed consent form and no ethical issues are known to exist with the use of placenta-derived cells. Placenta collection and use is approved by the Israeli medical center Ethics Committees (protocol number PLC-001-03 MOH reference number: 302102218).

### 2.2. Isolation of Mesenchymal Stromal Cells from the Amniotic Membrane

Human term placentas were obtained from healthy women with informed consent after vaginal delivery or caesarean section and processed immediately. Cells were isolated as previously described [41]. The amnion was manually separated from the chorion, washed in saline solution containing 100 U/mL penicillin and 100 μg/mL streptomycin (catalog number P0781), and cut into small pieces. Amnion fragments were digested at 37 °C for 9 min with 2.5 U/mL dispase (catalog number 734–1312 from VWR, Radnor, PA, USA), and then transferred to RPMI complete medium (catalog number R0883) composed of RPMI 1640 medium supplemented with 10% heat-inactivated fetal bovine serum (FBS) (catalog number F9665), 1% penicillin, and streptomycin (herein referred to as P/S), and 1% L-glutamine (catalog number G7513) (all from Sigma Aldrich, St. Louis, MO, USA). Afterward, the fragments were treated with 0.94 mg/mL collagenase (catalog number 11088793001) and DNase I (catalog number 11284932001) (both from Roche, Basel, Switzerland) for approximately 2.5–3 h at 37 °C. Resulting cell suspensions were centrifuged at low g. The supernatant was filtered through a 100-μm cell strainer (catalog number CLS431752 from BD Falcon, Bedford, MA, USA) and the cells were collected by centrifugation. Freshly isolated (p0) are referred to as hAMSC and were expanded until passage 1 (p1) by plating at a density of 10^4^/cm^2^ in Chang medium C (catalog number 12400080 from Irvine Scientific, Santa Ana, CA, USA) supplemented with 2 mM L glutamine at 37 °C in the incubator at 5% CO_2_.

### 2.3. Placental Expanded (PLX) Cells

PLX is an allogeneic ex-vivo placental expanded adherent stromal cell product obtained from Pluristem LTD in the GMP compliant facilities located at Haifa Israel. The mesenchymal-like stromal cells, referred to as adherent stromal cells, are derived from the full-term human placenta collected from healthy women undergoing an elective caesarean section and expanded using plastic adherence on tissue culture dishes. This was followed by three-dimensional growth on carriers in a bioreactor, as previously described [42,43,44]. The manufacturing process consists of two stages. In the first stage, the cells are digested from the placenta and expanded in 2-dimensional (2D) cell growth for several passages after which the cells are concentrated and cryopreserved to produce vials containing the Intermediate Cell Stock (ICS). In the second stage of the production, one vial of ICS is further cultured to produce the final PLX-PAD product. After thawing, the ICS is cultured in 2D for additional passages until the culture reaches 60–90% confluency and then transferred to bioreactors for a final culture in controlled 3D-expansion on carriers. The final PLX-PAD drug product is immediately formulated, filled in vials, and cryopreserved. The growth stage at the bioreactor is automatically controlled to keep ideal growth conditions such as Dissolved Oxygen (DO) at 70%. From each placenta, several ICS vials are being produced and, after thawing each ICS vial, can produce one PLX-PAD batch. The overall population doubling level of the cells does not exceed 25 doublings.

The results obtained from maternal-derived PLX-PAD cells are representative of the cumulative data obtained from two different batches of PLX-PAD cells supplied by Pluristem LTD, Israel. For fetal PLX-R18 cells, one batch was used for all experiments.

### 2.4. Analysis of PLX Cells and hAMSC Phenotype

Both maternal (PLX-PAD) and fetal (PLX-R18 and hAMSC) cell populations (hereafter, collectively referred to as “MSC”) were analyzed by flow cytometry for the expression of CD90 (clone 5E10), CD105 (clone 266), CD73 (clone AD2), CD13 (clone L138), CD45 (clone HI30), CD66b (clone G10F5), CD14 (clone MΦP9), CD34 (clone 581/CD34), CD107a (clone H4A3), CD146 (clone P1H12), CD140b (clone 28D4), CD40 (clone 5C3), CD80 (L307.4), CD86 (clone FUN-1), CD95 (clone DX2), CD178 (clone NOK-1), CD273 (clone MIH18), CD274 (clone MIH1), CD200 (MRC OX-104), CD324 (clone 67A4), CD326 (clone HEA-125), Galectin-9 (clone REA435), B7H4 (clone MIH43), HLA-ABC (clone G46-2.6), HLA-DR (clone TU36), HLA-DQ (clone TU169), HLA-DM (clone MaP.DM1), and HLA-G (clone MEM-G9). All antibodies were purchased from BD Biosciences (BD Biosciences, Franklin Lakes, NJ, USA), except for HLA-G, which was purchased from Serotec-Bio-Rad, (Hercules, CA, USA), and CD324, CD326, and Galectin-9, which were purchased from Miltenyi Biotec (Bergisch Gladbach, Germany). Dead cells were gated out by E-Fluor 780 (catalog number 65-0865-14, Thermofisher, Waltham, MA, USA) staining. Surface staining was carried out at 4 °C for 30 min by using a standard procedure.

The intracellular staining for human leukocyte antigen (HLA)-DM and Galectin 9 was performed upon fixation and permeabilization following with BD Cytofix/Cytoperm (catalog number 554714), BD Biosciences, Franklin Lakes, NJ, USA). Cells were then incubated with anti-HLA-DM or anti-Galectin 9.

Antigen expression was detected using FACSAria III (BD Biosciences) and data were analyzed with FCS express v5 (De Novo Software, Los Angeles, CA, USA).

### 2.5. Analysis of T Cell Proliferation

Human peripheral blood mononuclear cells (PBMC) were obtained from heparinized whole blood samples donated by healthy subjects (*n* = 24) using density gradient centrifugation (Histopaque 1077, catalog number 10771, Sigma-Aldrich, St. Louis, MO, USA).

To study the effect of maternal-derived and fetal-derived cells on PBMC proliferation upon stimulation with anti-CD3 OKT3 and anti-CD28 mAbs, MSC were seeded in 96 well plates and given the chance to adhere overnight. The day after, MSC were irradiated at 30Gy and 1 × 10^5^ allogeneic PBMC were added to each well. The co-culture was performed with three different PBMC:MSC ratios (1:1, 1:0.5 and 1:0.1) and stimulated or not (for the basal activation of the PBMC) with 0.125 μg/mL of CD3 mAbs (CD3 Orthoclone OKT3, catalog number L04AA02, Janssen-Cilag, Neuss, Dusseldorf, Germany) and 7 μg/mL of CD28 mAbs (CD28 soluble anti-CD28.2, catalog number 555725, BD Biosciences). Cell proliferation was assessed three days after stimulation by adding EdU 16–18 h before harvesting, as previously described (catalog number C10425, Life Technologies, Carlsbad, CA, USA) [45]. Cells were stained with E-Fluor 780 (catalog number L34975, Thermofisher) for the exclusion of dead cells and with anti-CD45 (clone HI30), anti-CD3 (clone UCHT1), anti-CD4 (clone SK3), anti-CD8 (clone SK1), anti-CD56 (clone N901), and anti-CD14 (clone MφP9). All antibodies were purchased from BD Biosciences (BD Biosciences, Franklin Lakes, NJ, USA) except for CD56, which was purchased from Beckman Coulter. Cells were acquired at FACSAria III (BD Biosciences) and the percentage of proliferating EdU-positive cells was analyzed with FCS express v5 (De Novo Software, Los Angeles, CA, USA).

### 2.6. Degranulation and Cytotoxic Marker Expression

To study the capacity of maternal-derived and fetal-derived MSC to modulate the expression of cytotoxicity markers on PBMC activated with anti-CD3 and anti-CD28 mAbs. MSC were seeded in 96 well plates and given a chance to adhere overnight. The day after MSC were irradiated at 30Gy and allogeneic PBMC were added to each well. The co-culture was performed with two different PBMC:MSC ratios (1:1, 1:0.5) chosen based on the results previously obtained in the proliferation inhibition tests. Lymphocytes were stimulated or not (for the basal activation of the PBMC) with CD3/CD28 mAbs. Cytotoxic activity was assessed three days after stimulation. PBMC were stimulated for 4 h with 10 µg/mL Phorbol Myristate Acetate (PMA) (catalog number P1585) and 6 µg/mL Ionomycin (catalog number I0634, both from Sigma-Aldrich). After 1 h and 15 min, 30 μg/mL of Brefeldin A (catalog number B7651, Sigma Aldrich) was added.

For degranulation assays, cultured PBMC were incubated in the presence of anti-CD107a (clone H4A3) monoclonal antibody (mAb) with a Golgi stop (catalog number 554724, both from BD Biosciences) directly added in parallel with PMA and Ionomycin. CD107a surface expression on effector cells was assessed after 4 h.

To detect spontaneous degranulation or constitutive expression of cytokines/cytotoxic effectors, an unstimulated control condition was included.

Cells were then stained with E-Fluor 780 (Thermofisher) for the exclusion of dead cells and with anti-CD3, anti-CD8, anti-CD45, anti-CD14, and anti-CD56 for the surface staining. CD4^+^ T lymphocytes are represented by CD45^+^CD3^+^CD8^−^ cells. The intracellular staining for Perforin (clone δG9), Granzyme B (GrzB) (clone GB11), and IFN-γ (clone B27) was performed upon fixation and permeabilization with BD Cytofix/Cytoperm (all from BD Biosciences). All the antibodies were purchased from BD Biosciences (BD Biosciences). Cells were acquired at FACSAria III (BD Biosciences) and the analyzed with FCS express v5 (De Novo Software, Los Angeles, CA, USA).

### 2.7. Phenotype of CD4^+^ T Helper (Th) and T Regulatory (Treg) Subsets

The phenotypes of the different Th and Treg subsets were assessed by a panel of specific surface markers for the expression of the transcriptional factor FoxP3. After 6 days of a co-culture with maternal or fetal-derived MSC performed at the same PBMC:MSC ratios used for the degranulation and cytotoxicity marker assay, CD3/CD28 PBMC were collected and centrifuged at 300 g for 5 min. Cells were stained with E-Fluor 780 (Thermofisher) for the exclusion of dead cells. The surface staining was performed using antibodies for CD3, CD4, CD45RA (clone HI100), CD196 (clone 11A9), CD183 (clone 1C6/CXCR3), CD194 (clone REA279), CD161 (clone DX12), CD25 (clone M-A25), and CD127 (clone MB15-18C9), which all came from BD Biosciences. The intracellular staining for FoxP3 (clone 259D/C7) was performed upon fixation and permeabilization with BD Cytofix/Cytoperm (BD Biosciences). Cells were then incubated with anti-FoxP3 antibody, acquired at FACSAria III (BD Biosciences), and Th/Treg subsets analyzed with FCS express v5 (De Novo Software, Los Angeles, CA, USA).

### 2.8. Analysis of Monocyte Differentiation toward Antigen Presenting Cells

Monocytes (Mo) were purified from PBMC by positive selection using anti-CD14-coated microbeads and MACS^®^ separation columns (catalog number 130-250-201, Miltenyi Biotec). Monocyte-derived dendritic cells (DC) were obtained as previously described [46], with modification. DC were obtained from allogeneic purified Mo (5 × 10^5^ cells) seeded in 48-well plates for four days (Corning) in the presence of recombinant human IL-4 (catalog number 204IL, R&D Systems, Minneapolis, MN, USA) (50 ng/mL) and granulocyte macrophage-colony stimulating (GM-CSF, catalog number 130-093-862, Miltenyi Biotec) (50 ng/mL) in 0.5 mL RPMI 1640 complete medium (Sigma Aldrich). Complete maturation was reached by adding lipopolysaccharide (LPS) (catalog number L4516, Sigma Aldrich) 0.1 μg/mL for two days.

Monocyte-derived M1 macrophage cells were obtained as previously described in Reference [45].

To analyze the effect of MSC on monocyte differentiation, MSC were seeded in RPMI complete medium and given the chance to adhere overnight. The next day, MSC were gamma-irradiated at 30Gy and Mo were added. The co-culture was performed with two different Mo:MSC ratios (1:0.4 and 1:0.2) for both DC and M1 macrophages, as described previously [46,47,48,49].

mDC and M1 macrophages were collected after 6 days of differentiation. The phenotypic profile was investigated by flow cytometry. Prior to the surface staining, cells were stained with E-Fluor 780 (Thermofisher) for the exclusion of dead cells. Then cells were surface stained with anti-CD45, anti-CD80 (clone L307.4), anti-CD1a (clone HI149), anti-CD163 (clone GHI/61), anti-CD209 (clone DCN46), anti-CD197 (clone 3D12), and anti-CD14 antibodies (purchased from BD Biosciences).

### 2.9. Cytokine/Chemokine Analysis

Cytokine/chemokine levels were measured in supernatants collected from PBMC stimulated with CD3/CD28 mAbs. 1 × 10^5^ PBMC were stimulated with CD3/CD28 mAbs and cultured in 96 well plates in the absence or presence of PLX-PAD, PLX-R18, or hAMSC cells at a PBMC:MSC ratio of 1:1. The supernatant was collected after 6 days and stored at −80 °C. Supernatants from PLX-PAD, PLX-R18, or hAMSC cells cultured alone were all included as controls. Each supernatant was thawed right before use in cytokine/chemokine assays. A multiplex bead-based immunoassay (BD CBA Flex Set system from BD Biosciences) was used to determine the levels of human IFN-γ (catalog number 560111), TNFα (catalog number 560112), IL-4 (catalog number 558262), IL-5 (catalog number 557288), IL-13 (catalog number 558450), IL-10 (catalog number 558274), TGF-β1 (catalog number 560429), IL-17A (catalog number 560383), Granzyme-A (GrzA) (catalog number 560299), Granzyme-B (GrzB) (catalog number 560304), Regulated on Activation, Normal T Cell Expressed and Secreted (RANTES/CCL5) (catalog number 558324). Samples were processed, according to the manufacturer’s instructions, acquired using a FACSAria III (BD Biosciences) and analyzed using FCAP Array software (BD Biosciences).

### 2.10. Analysis of Immunogenicity

To study the capacity of maternal-derived and fetal-derived MSC to induce PBMC proliferation, MSC were seeded in RPMI complete medium and left to adhere overnight. The next day, cells were irradiated (30Gy) and an allogeneic responder PBMC were added. Five different PBMC:MSC ratios (1:1, 1:0.5, 1:0.25, 1:0.125, and 1:0.0625) were tested. All cultures were carried out in triplicate using round-bottomed 96-well tissue culture plates (Corning) in a final volume of 200 µL of RPMI complete medium. As a positive control for PBMC activation, allogeneic PBMC and mDC cells were additionally used as stimulators and added at the same ratios used to test the immunogenic properties of MSC. MSC, allogeneic PBMC, and mDC were irradiated to ensure that any proliferation observed could be attributed solely to the proliferation of responder lymphocytes. Proliferation of PBMC was assessed after 6 days by adding [3H]-thymidine (0.7 μCi per well, catalog number NET027250UC, PerkinElmer) for 16–18 h. Cells were then harvested with a Filtermate Harvester, and thymidine incorporation was measured using a microplate scintillation and luminescence counter (Top Count NXT), which are both from PerkinElmer (PerkinElmer Waltham, MA, USA).

### 2.11. Statistical Analysis

The data are displayed as box plots and histograms with Tukey variations. The parameters were compared using one-way analysis of variance. Data are representative of at least four independent experiments. Statistical analysis was performed using Prism 6 (GraphPad Software, La Jolla, CA, USA). A *p*-value lower than 0.05 was considered statistically significant.

## 3. Results

### 3.1. Immunophenotype of Maternal and Fetal Cells

**Figure 1:**
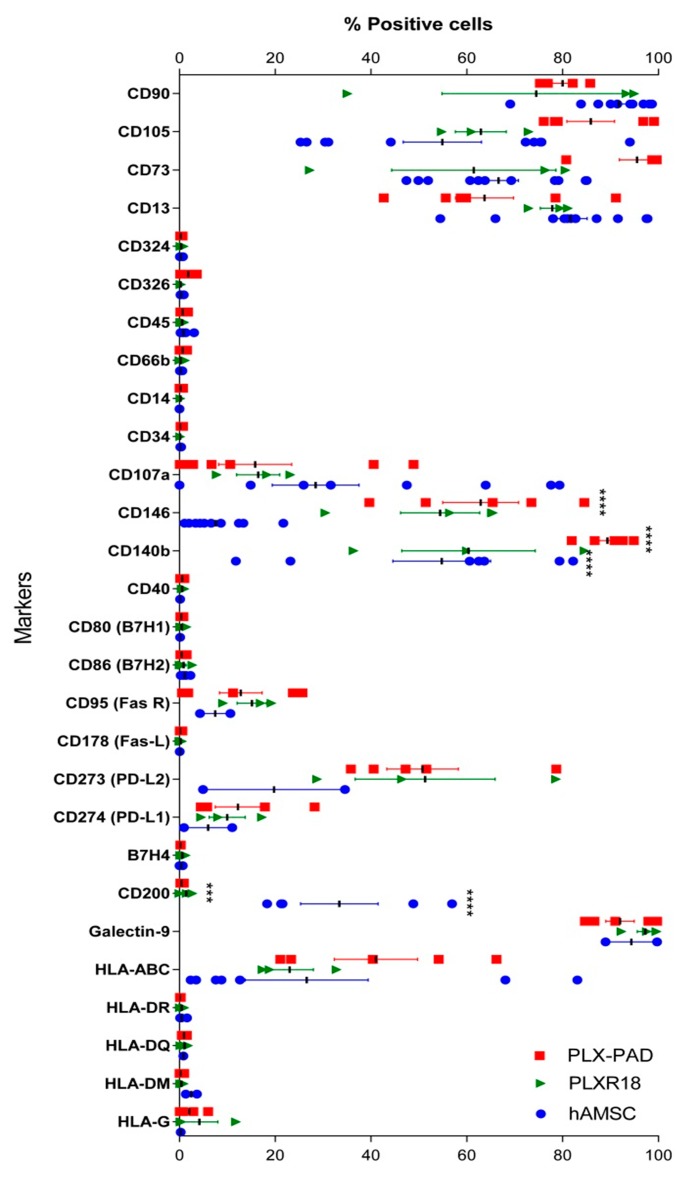

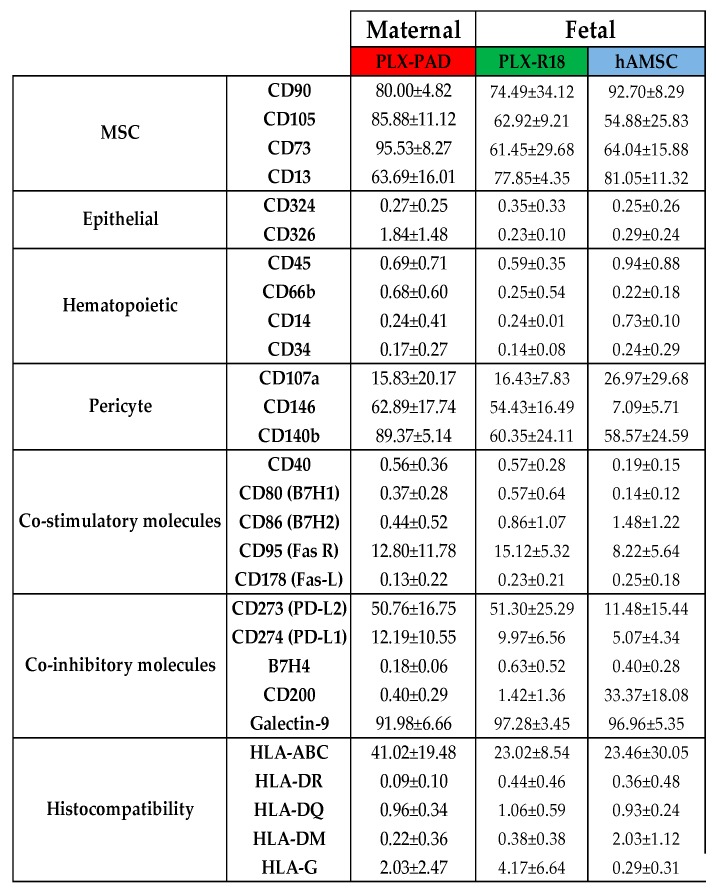
Phenotype analysis of cells derived from different batches of maternal (PLX-PAD R06 and R08) and fetal (PLX-R18 and hAMSC) placental tissues. Immune phenotype screening of the three different cell populations. Phenotype was analyzed by flow cytometry and data are presented as mean ± SD (*** *p* < 0.001, **** *p* < 0.0001). Results were obtained from biological replicates obtained from different experiments (*n* ≥ 3 individual experiments).

We first analyzed the immunophenotype of maternal (PLX-PAD) and fetal (PLX-R18 and hAMSC) cells considering the expression of a panel of CD markers (Figure 1). More specifically, all three cell populations expressed typical MSC markers including CD13, CD73, CD105, and CD90, and had a low/absent expression of hematopoietic markers (CD14, CD34, and CD45) and epithelial markers (CD324 and CD326). Moreover, both maternal and fetal populations expressed HLA-ABC, but lacked the expression of the different HLA-II isoforms (HLA-DR, HLA-DQ, HLA-DM). It had very low expression of HLA-G, where the latter has a documented role in fetal-maternal tolerance [50].

Furthermore, we evaluated the expression of antigen presenting cells (APC) co-stimulatory molecules (CD80, CD86, CD40, CD95) and co-inhibitory molecules (CD273, CD274, B7H4, CD200, Galectin-9). Both maternal and fetal cell populations did not express co-stimulatory markers, with the exception of CD95 (stimulatory) [51,52,53] that was expressed by all three populations. On the other hand, co-inhibitory markers such as CD273 (PD-L2), CD274 (PD-L1), and Galectin-9 were expressed by all three populations, where the latter was highly expressed (>70%), (Figure 1). Lastly, we confirmed the differential expression of the CD200 inhibitory ligand as reported by others [33,54], whereby maternal cells were negative for this ligand and the two fetal cell populations had variable expression. In this case, hAMSC moderately expressed CD200 (33.3% ± 18.8) and PLX-R18 had a very low CD200 expression (1.4% ± 1.36), (Figure 1).

### 3.2. Maternal and Fetal Cells Differently Impact the Proliferation of T Lymphocytes

**Figure 2:**
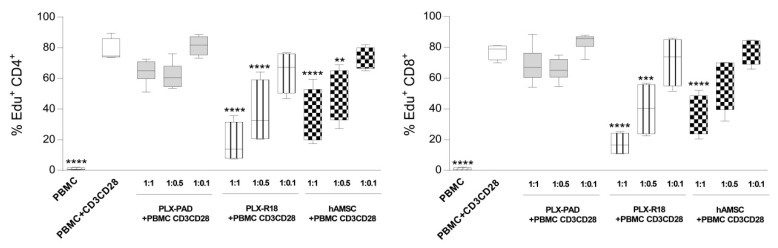
Effect of placental cells on T lymphocyte proliferation. Allogeneic PBMC (1 × 10^5^) were stimulated with anti-CD3/CD28 antibodies in the presence of decreasing ratios of maternal (PLX-PAD)-derived or fetal (PLX-R18 or hAMSC)-derived cells. Cells were cultured for three days, and proliferation was assessed by Edu (ethynyldeoxyuridine) incorporation added during the final 18 h of culture. Results are presented for both CD4^+^ and CD8^+^ T lymphocyte cell subsets and are expressed as a percentage of cell proliferation. PBMC stimulated with anti-CD3/CD28 mAbs constitute the positive control while PBMC alone represent the basal level of proliferation. Results are displayed as mean±SEM (** *p* < 0.01, *** *p* < 0.001, **** *p* < 0.0001 versus control PBMC+ CD3CD28), *n* ≥ 4 individual experiments.

We next evaluated the capacity of maternal-derived or fetal-derived placenta cells to inhibit T-cell proliferation. Approximately 80% of CD4^+^ (79.5 ± 7.53%; Figure 2, left panel) and CD8^+^ (81 ± 9.8%, Figure 2, right panel T cells (range 60–85%, *n* = 4) proliferated upon stimulation with CD3/CD28 mAbs. Both CD4^+^ and CD8^+^ T-cell proliferation were modestly reduced by maternal cells. In the presence of a 1:1 ratio of maternal cells, CD4^+^ T cell proliferation was 67.7 ± 9.6% and CD8^+^ T cell proliferation was of 71.46 ± 13.3% (Figure 2). On the other hand, fetal cells significantly reduced T-cell proliferation triggered through the polyclonal stimulus CD3/CD28 mAbs in a dose-dependent manner (Figure 2). CD4^+^ T cell proliferation was significantly reduced by fetal-derived cells (proliferation at 1:1 ratio of 17.17 ± 13.15% for PLX-R18, 36.3 ± 16.1% for hAMSC). Similar results were observed concerning the proliferation of CD8^+^ T cells when co-cultured with fetal cell populations (proliferation at 1:1 ratio of 17.17 ± 7.15% for PLX-R18, 40.7 ± 15.5% for hAMSC).

### 3.3. Maternal and Fetal Cells Affect T Lymphocyte Functions and Reduce the Expression of Cytotoxicity Markers

**Figure 3:**
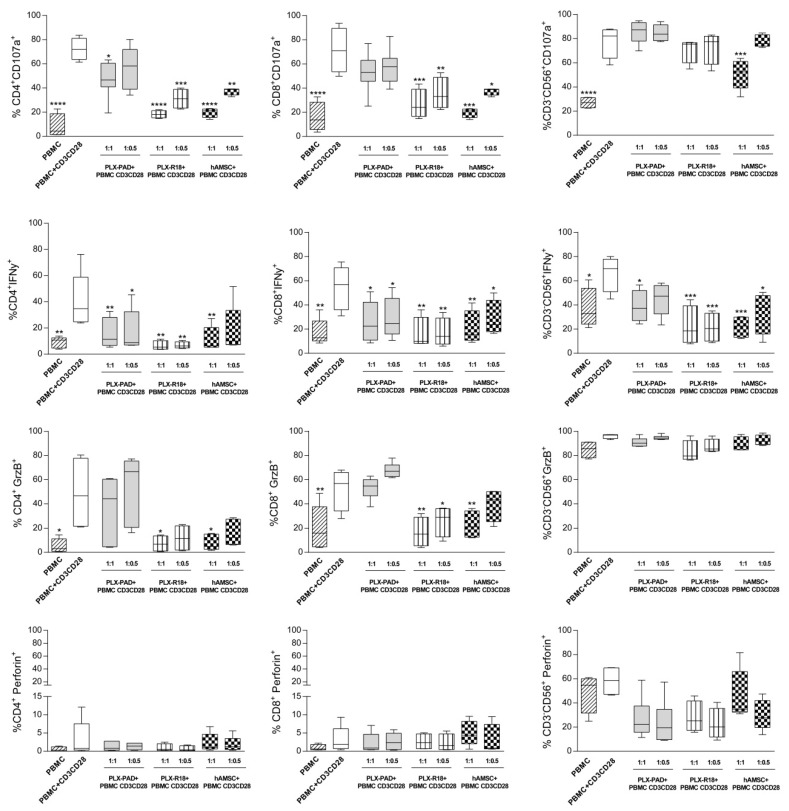
Cytotoxic activity marker expression by T lymphocytes and NK cells after interaction with PLX-PAD cells. Allogeneic PBMC were incubated with anti-CD3/CD28 antibodies in the presence of 2 ratios (1:1 and 1:0.5) of maternal (PLX-PAD)-derived or fetal (PLX-R18 or hAMSC)-derived cells. After two days of culturing, PBMC were activated with PMA+Ionomycin and Golgistop was added 1 h later and, 4 h later, the cells were collected and stained. The frequency of CD107a, IFN-γ, Granzyme B (GrzB^+^), and Perforin positive cells (Perforin^+^) within the CD4^+^, CD8^+^ T cell, and CD3^−^CD56^+^ NK cell population was assessed by flow cytometry. PBMC alone or incubated with anti-CD3/CD28 mAbs only were used as controls. Results are displayed as mean ± SEM (* *p* < 0.05, ** *p* < 0.01, *** *p* < 0.001, **** *p* < 0.0001 versus control PBMC+ CD3/CD28), *n* ≥ 4 individual experiments.

In order to evaluate the ability of maternal and fetal cells to trigger and/or modulate the cytotoxic activity, we evaluated the expression of cytotoxicity markers CD107a (lysosome-associated membrane protein 1), Granzyme-B (GrzB), Perforin, and the inflammatory cytokine IFN-γ expressed by CD4^+^ and CD8^+^ T cells, and CD3^−^CD56^+^ NK cells in PBMC activated by CD3/CD28 mAbs. Since, in our previous experiments, the lower PBMC:MSC ratio (1:0.1) tested was ineffective to reduce T lymphocyte proliferation; we excluded this ratio in the subsequent analysis. CD107a was strongly expressed by CD3/CD28-stimulated PBMC (69.7% ± 6.6 for CD4^+^, 75.6 ± 18.7 for CD8^+^, 81.5% ± 8.3 for NK cells), (Figure 3). At the highest ratio (1:1) tested, PLX-PAD cells were able to significantly reduce cytotoxic degranulation (evaluated as the inhibition of CD107a surface expression) on CD4^+^ T cells (47.7% ± 13.3). A reduction of CD107a was also seen on CD8^+^ T cells (52.7% ± 15.2), (Figure 3) even if not statistically significant. Instead, no effects were observed on NK cells (85.9% ± 9.4), (Figure 3). Moreover, the inflammatory cytokine IFN-γ was inhibited by the PLX-PAD on both CD4^+^ (15.7% ± 11.4) and CD8^+^ T cells (26.4% ± 16.3), and also on NK cells (38.7% ± 12.3), (Figure 3). Lastly, the expression of Granzyme-B and Perforin were not significantly affected by maternal cells, but fetal-derived cells showed a stronger inhibitory effect on cytotoxic activity of lymphocytes (Figure 3). Both PLX-R18 and hAMSC significantly reduced the expression of CD107a on CD4^+^ (18.2% ± 3.1 for PLX-R18 and 19.8% ± 4.0 for hAMSC) and CD8^+^ (26.6% ± 12.2 for PLX-R18 and 8.8% ± 14.9 for hAMSC) T cells and, in the case of hAMSC, also on NK cells (51.6% ± 14.0). Similar to maternal cells, fetal-derived cells also inhibited the expression of IFN-γ on both CD4^+^ (6.5% ± 3.6 for PLX-R18 and 11.5% ± 9.4 for hAMSC) and CD8^+^ (15.9% ± 13.4 for PLX-R18 and 20.5% ± 14.4 for hAMSC) T cells, and on NK cells (22.3% ± 18.2 for PLX-R18 and 21.9% ± 9.0 for hAMSC). In addition, fetal-derived cells also reduced the expression of Granzyme-B on CD4^+^ (7.1% ± 6.8 for PLX-R18 and 8.8% ± 7.3 for hAMSC), and CD8^+^ (16.5% ± 12.2 for PLX-R18 and 22.6% ± 11.6 for hAMSC) T cells, but not on NK cells (Figure 3). Overall, our results suggest that both maternal and fetal cells can impair the cytotoxic activity of CD4^+^ and CD8^+^ T lymphocytes and NK cells, and the strongest inhibitory effects were obtained with fetal cells.

### 3.4. Maternal and Fetal Cells Inhibit Th1 Priming and Strongly Induce Pro-Regenerative Th22 and T Regulatory Cell Subsets

We have previously demonstrated that hAMSC are able to modulate different lymphocyte subsets [45]. Herein, we assessed the effects of maternal and fetal-derived cells on T helper (Th) Th1, Th17, Th1/Th17, Th2, Th22, and T regulatory (Treg) subset polarization.

**Figure 4:**
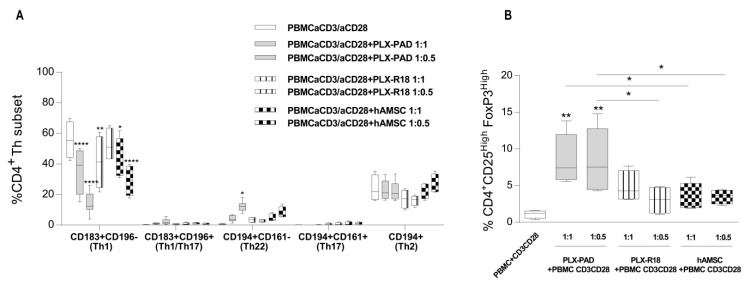
Effect of maternal and fetal derived MSC on Th1/Th2 and Treg polarization. Allogeneic PBMC were stimulated with anti-CD3/CD28 mAbs and co-cultured with MSC for six days. (**A**) Th1 (CD183^+^CD196^−^), Th1/Th17 (CD183^+^CD196^+^), Th22 (CD194^+^CD161^−^), Th17 (CD194^+^CD161^+^), and Th2 (CD194^+^) phenotypes were evaluated by flow cytometry at day 7 and expressed as a percentage of CD4^+^CD45RA^−^ gated cells. (**B**) Induction of Treg was evaluated by flow cytometry after six days of co-culture and it is displayed as a percentage of CD45RA^−^ CD25^hi^FoxP3^hi^ cells. Results are represented as mean±SEM (* *p* < 0.05, ** *p* < 0.01, **** *p* < 0.0001), *n*≥ 4 individual experiments.

Upon activation with CD3/CD28 mAbs, control PBMC highly expressed Th1 subset markers (CD183^+^CD196^+^) (55.6% ± 12.08 gated on CD4^+^CD45RA^−^ T lymphocyte, Figure 4A). Furthermore, the percentage of Treg cells was approximately 1.13% ± 0.53 (Figure 4B). When activated, PBMC were co-cultured with maternal-derived or fetal-derived cells, there was a strong and significant reduction in Th1 polarization, and more potent effects were observed with maternal cells at both PBMC:MSC ratios tested (maternal PLX-PAD MSC 1:1 ratio: 35.5 + 14.02, 1:0.5:14.3 + 7.08 vs. 41.15 + 17.2 for PBMC:PLX-R18 1:1 and 42.6 + 13.3 for PBMC:hAMSC 1:1), (Figure 4A). We observed that both maternal and fetal cells trigger Th22 polarization (0.50% ± 0.44 for the control condition to 4.70% ± 3.45 for PLX-PAD cells and 3.43% ± 1.73 and 4.69 + 2.37 for PLX-R18 and hAMSC, respectively). The percentage of Treg cells increased (1.13% ± 0.53 for stimulated control PBMC to 8.73% ± 3.3 for co-culturing with maternal PLX-PAD, 4.83% ± 2.10 with PLX-R18, and 3.51% ± 0.97 with hAMSC), (Figure 4B). Th1/Th17, Th17, and Th2 polarization was unaffected (Figure 4A). Altogether, these findings suggest that both maternal and fetal cells reduce polarization toward the inflammatory Th1 cell subset and trigger polarization toward the pro-regenerative and anti-inflammatory Th22 and Treg cell subset.

### 3.5. Maternal and Fetal MSC Affect the Expression of Th-Cytokines

In order to provide further insight and to potentially confirm the ability of maternal and fetal cells to impact Th subset polarization observed after co-culture with either maternal and fetal cells, we evaluated a panel of cytokines specifically expressed by different T cell subsets: Th1 (IFN-γ, TNFα), Th2 (IL-4, IL-5, IL-13), Th17 (IL-17A), Treg (IL-10, TGFβ), and cytotoxic cells (GrzB, GrzA, RANTES), (Table 1). The amount of cytokines and chemokines produced and released by PLX-PAD, PLX-R18, and hAMSC alone were also measured (Supplementary Table S1).

We observed that, in the presence of either maternal or fetal cells, the secretion of Th1 inflammatory cytokines IFN-γ and TNFα was strongly reduced, which confirms the data previously observed and indicates the reduction of the Th1 subset polarization.

The analysis of the expression of the canonical Th2 subset cytokines revealed that the expression of IL-4 was barely detectable. IL-5 decreased in the presence of either maternal and fetal MSC, while IL-13 decreased only with fetal MSC. The expression of Th17-related cytokine IL-17A resulted in a decrease by maternal PLX-PAD cells while no effect was observed when the co-culture was performed with fetal derived MSC. The results obtained from the analysis of Treg cell-related cytokines IL-10 demonstrated no detectable differences between the control (PBMC+ CD3/CD28) and the PBMC co-cultured with either maternal or fetal MSC. The expression of TGFβ1 was, instead, significantly higher in all the three co-culture conditions compared to the control, but the results could be due to, at least in part, the high amount of TGFβ1 produced and released by both maternal and fetal MSC (Supplementary Table S1).

Lastly, we also analyzed the production of cytotoxic GrzB, GrzA, and RANTES. The production of GrzB, whose intracellular expression was not down-regulated in the presence of PLX-PAD cells on day 3 (Figure 3), decreased in comparison to the control condition at day 6. Again, fetal cells resulted in more effective reduction of GrzB production than maternal PLX-PAD. GrzA also showed a trend of inhibition in PBMC activated in the presence of both fetal and maternal cells. Secretion of RANTES (regulated upon activation, normal T cell expressed and secreted) was inhibited by fetal cells (both PLX-R18 and hAMSC) and not by a maternal PLX-PAD.

### 3.6. Maternal and Fetal Cells Inhibit Monocyte-Derived Antigen Presenting Cell (APC) Differentiation

**Figure 5:**
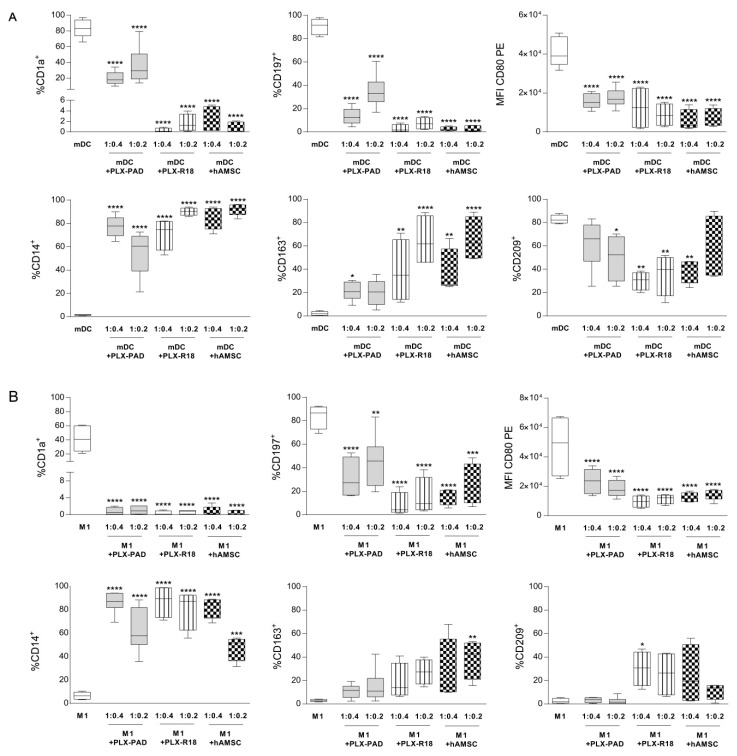
Effect of placental cells on the monocyte to APC differentiation. Phenotypic analysis of isolated CD14^+^ monocytes differentiated into (**A**) mature dendritic cells (mDC) or (**B**) M1 macrophages in the absence or presence of maternal (PLX-PAD)-derived or fetal (PLX-R18 or hAMSC)-derived cells. mDC differentiation was carried out by incubating the cells with GM-CSF+IL-4 for four days followed by two days of LPS treatment. (**A**). M1 macrophages were obtained by incubating CD14^+^ monocytes with GM-CSF for four days, which is followed by IFN-γ+LPS for another two days. (**B**). At the end of the culture period, expression of CD1a, CD14, CD197, CD163, CD80, and CD209 was evaluated by flow cytometry. Results are presented as a percentage of expression (except for CD80 where MFI is displayed), and are shown as mean ± SEM (* *p* < 0.05, ** *p* < 0.01, *** *p* < 0.001, **** *p* < 0.0001 versus control mDC or M1), *n* ≥ 4 individual experiments.

Next, we analyzed if maternal (PLX-PAD) or fetal-derived cells (PLX-R18 and hAMSC) were able to directly impact monocyte (Mo) differentiation toward APC. Purified monocytes were differentiated to M1 macrophages or mature DC (mDC) in the absence (control) or presence of maternal and fetal-derived cells at two different ratios (Mo:MSC = 1:0.4 or 1:0.2). As previously reported [45] and as shown in Figure 5A,B, during M1 differentiation, monocytes lost the monocytic marker CD14 (6.54% ± 3.12) and acquired the expression of CD1a (41.85% ± 18.13), the chemokine receptor CCR7 (CD197) (83.7% ± 10.3), and the co-stimulatory molecule CD80 (38,038 ± 13,357). The expression of CD163 (2.9 ± 0.97) and DC-SIGN (CD209) (2.44% ± 2.18) was absent. Similar to M1 macrophages, mDC expressed CD1a (83% ± 11.23), CD197 (90.2% + 6.87), and CD80 (36,353 ± 4392), whereas they lacked CD14 (1.56% ± 0.4) and CD163 (2.8 ± 1.36). Moreover, mDC were characterized by the expression of DC-SIGN (CD209) (82.6% ± 4.54), which is a marker absent on M1 macrophages.

We observed that both maternal (PLX-PAD) and fetal-derived cells (PLX-R18 and hAMSC) strongly blocked the differentiation of mDC and M1 at both ratios tested (Figure 5 panels A and B, respectively). There was a reduction in the expression of the differentiation markers CD1a (for mDC: CD1a = 21.3 + 11 for maternal PLX-PAD, 0.3 + 0.4 for PLX-R18, and 2.3 + 2.29 for hAMSC, Figure 5A, for M1 macrophages: CD1a = 2.09 ± 4.80 for maternal PLX-PAD, 0.3 + 0.51 for PLX-R18, and 4.3 + 8.6 for hAMSC, Figure 5B) and CD197 (for mDC: CD197 = 17.7 ± 13.13 for maternal PLX-PAD, 3.43 ± 3.7 and 2.6 ± 1.72 for fetal PLX-R18 and hAMSC, respectively, Figure 5A, for M1 macrophages: maternal PLX-PAD = 30.12 ± 14.7, PLX-R18 = 8.43 ± 10.38, hAMSC = 15.85 ± 6.95 for hAMSC, Figure 5B), and the expression of the undifferentiated monocytic marker CD14 was maintained (for mDC: 86% ± 8.45 for maternal PLX-PAD, 87% ± 13.7 for PLX-R18 and 82.4% ± 8.3 for hAMSC, for M1 macrophages: maternal PLX-PAD = 86.16 ± 8.45, PLX-R18 = 87 ± 13.71, hAMSC = 82.4 ± 9.33). Furthermore, as previously observed [46], the percentage of CD80^+^ cells remained unaltered compared to control mDCs or M1 macrophages, while the intensity of CD80 expression changed. The median fluorescence intensity of the co-stimulatory molecule CD80 was reduced (for mDC: 14,793 ± 3246 for maternal PLX-PAD, 12,379 ± 10,836 for PLX-R18, and 7216 ± 6054 for hAMSC, Figure 5A, for M1 macrophages: maternal PLX-PAD = 19,258 ± 8370, PLX-R18 = 9493 ± 4034, hAMSC = 13,302 ± 3033, Figure 5B). The expression of CD209 was induced during M1 differentiation by fetal cells (maternal PLX-PAD = 5.12 ± 6.25, PLX-R18 = 30.2 ± 14.77, hAMSC = 24.34 ± 25.65, Figure 5A), and reduced during mDC differentiation (61.7% ± 20.9 for maternal PLX-PAD, 30.4% ± 3.8 for PLX-R18, and 37.03% ± 11.36, Figure 5B). In addition, during mDC and M1 differentiation, in the presence of maternal and fetal-derived cells, we observed the increased expression of CD163 (for mDC: maternal PLX-PAD = 26.46 ± 6.32 and for the fetal 64.5 ± 21.9, 70.3 ± 20.6 for PLX-R18 and hAMSC, respectively, Figure 5A), (for M1 macrophages: 2.9 ± 0.97, for maternal PLX-PAD = 10.88 ± 5.57 and for the fetal = 18.7 ± 15.39 for PLX-R18 and 38.5 ± 16.6 for hAMSC). The intermediate expression of CD209 and the expression of CD163 indicate that maternal and fetal cells interfere with the differentiation of monocytes toward M1 macrophages or mDC, and promote monocyte polarization toward anti-inflammatory M2 macrophage subsets.

### 3.7. Immunogenicity

**Figure 6:**
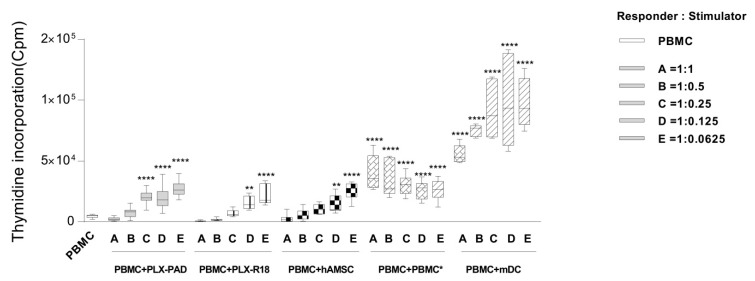
Immunogenicity of PLX-PAD cells. Responder PBMC (1 × 10^5^ cells per well) were plated in a 96-round well plate and co-cultured with gamma irradiated (30Gy) stimulator: allogeneic maternal and fetal MSC (PLX-PAD, PLX-R18, and hAMSC), PBMC*, and mDC. Responder and stimulator cells were co-cultured at five different ratios (A = 1:1, B = 1:0.5, C = 1:0.25, D = 1:0.125, and E = 1:0.0625) and mixed lymphocyte reaction (PBMC*) and mature dendritic cells (mDC) were used as positive controls of a complete immune response/activation. PBMC proliferation was assessed by H^3^-thymidine incorporation and data are expressed as count per minute (cpm) (** *p* < 0.01, **** *p* < 0.0001 versus control PBMC). *n* ≥ 7 individual experiments.

Lastly, we investigated the capacity of maternal and fetal cells to induce the proliferation of allogeneic PBMC in the absence of specific stimuli (herein referred to as immunogenicity). To demonstrate that the allogeneic PBMC were able to respond efficiently, these cells were also co-cultured with allogeneic PBMC by performing a mixed lymphocyte reaction (MLR), or with professional antigen-presenting cells (mDC) (Figure 6). At day 6, we observed that, at higher concentrations (1:1, 1:0.5), PLX-PAD, PLX-R18, and hAMSC did not induce PBMC proliferation. However, at the lower concentrations (1:0.25, 1:0.125 and 1:0.0625), maternal cells (and to a lower extent, fetal-derived cells) induced the proliferation of the responder PBMC comparable to that induced by allogeneic PBMC in a MLR. This was, however, lower than the proliferation induced by mDC.

## 4. Discussion

To dissect inherent features of MSC originated from distinct placental components, and given the well-documented ability of placental-derived MSC to modulate the immune response [25], herein, we compared the immune modulatory properties of MSC derived from maternal and fetal tissues of the human term placenta and included the systematic analysis of GMP preparations. The present study brings significant new insights into some of the placental MSC immune-modulatory properties by demonstrating in vitro that both maternal and fetal MSC (i) down-regulate T lymphocyte proliferation of PBMC stimulated with anti-CD3/CD28 mAbs, (ii) decrease the expression of cytotoxicity marker (CD107a, IFN-γ and GrzB) in anti-CD3/CD28-stimulated PBMC, and (iii) favor the differentiation of CD4^+^ T cells into subsets co-expressing high levels of CD25 and of FoxP3. In addition, maternal and fetal MSC affect Th polarization by skewing the T cell compartment towards Th22 cells, and (iv) strongly inhibit APC differentiation. Lastly, we observed that maternal and fetal cells did not present differences in their immunogenicity.

Positive expression of CD73, CD90, and CD105 and low/absent expression of hematopoietic markers (CD14, CD34, and CD45) have been suggested to define MSC [1,55]. Our data mostly corroborate these findings. However, in our studies, the positivity for CD73, CD90, and CD105 was lower than the recommended 95%. As reported for MSC from other sources [56], phenotype heterogeneity of placental-derived cells has been described, which was mainly induced by cell treatment (e.g., IFN-γ activation), different culture conditions, and the passage number [57,58]. The expression of CD105 is another variable reported in literature, ranging from low (4%) [59] to high (97%) expression [25]. Moreover, the expression of CD73, CD90, and CD105 increased during passages, and usually more than 90% of hAMSC expressed CD73, CD90, and CD105 from P2 to P4 [25,46]. hAMSC used in this study were from passage 1, which could account for the lower expression of these markers.

We observed that fetal cells possess enhanced anti-proliferative activity when compared to maternal cells. These results are in line with previous studies that have demonstrated that amnion and amniotic fluid-derived fetal MSC from second trimester placentas induced stronger anti-proliferative effects on PHA-activated lymphocytes and in MLR when compared to maternal MSC derived from the same tissues [32]. Similarly, other studies demonstrated that fetal-derived MSC had a higher suppressive activity on the proliferative response of PHA-stimulated PBMC when compared to maternal MSC [33].

Our observations herein expand on these studies whereby we not only demonstrate that fetal-derived cells have enhanced anti-proliferative properties versus T lymphocytes when compared to maternal-derived cells, but we also perform a detailed characterization of the immunomodulatory properties of both maternal and fetal cells by analyzing different T-cell subsets and by analyzing NK cell activation and monocyte differentiation toward APC.

First, we compared the ability of maternal and fetal derived MSC to modulate the immune response by evaluating how they impact the activation of T lymphocytes and NK cells through the analysis of different cytotoxicity markers and the expression of the inflammatory cytokine IFN-γ. Fetal-derived cells had a stronger ability to decrease the cytotoxic activity of both CD4^+^ and CD8^+^ T cells and, to a lesser extent, also NK cells, as shown by a significant reduction in the expression of the degranulation marker CD107a and IFN-γ. The ability of fetal-derived MSC, and, more specifically, hAMSC, to inhibit the cytotoxicity of NK cells against the K562 target has been previously demonstrated, and this was shown to be accompanied by a reduced expression of NCR (Natural cytotoxicity receptors) NKp30, NKp44, NKp46, NKG2D, and by the reduction of IFN-γ production [60]. As previously reported [54], we observed that OX-2 (CD200), which is a membrane glycoprotein that belongs to the immunoglobulin superfamily, was highly expressed by cells of fetal origin when compared to maternal-derived cells, and, particularly, by hAMSC. The CD200 receptor is capable of modulating the cytotoxic activity of NK cells [61,62] and could be one of the mechanisms responsible for the enhanced ability of fetal-derived MSC to inhibit NK cells when compared to maternal-derived MSC.

In addition, we observed differences between maternal-derived and fetal-derived cells in their ability to alter/impact T-cell subsets. Maternal-derived cells more strongly enhanced Treg subsets when compared to fetal-derived cells. Recent reports show that maternal MSC express a high level of indoleamine 2,3 dioxygenase (IDO) [32,63], which has been reported as one of the main factors responsible for the induction of Treg polarization [48,49,50]. Two other cytokines that play a relevant role in Treg induction are IL-10 [64] and TGFβ1 [65,66,67]. The low amount of IL-10 detected in hAMSC is in line with our previously published data [45,68]. Given that there were no relevant differences observed in the IL-10 production between maternal and fetal cells, this could suggest that IL-10 is likely not responsible for the different Treg polarization observed between maternal and fetal cells. This could be also the case for TGFβ1, where, the high expression observed by both maternal and fetal cells cannot account for the differences observed in the percentage of Treg cells observed. 

Furthermore, we observed that maternal cells more highly expressed CD73, which is an ectonucleotidase receptor responsible for the production of adenosine. This is known as a strong immune modulatory molecule involved in the modulation of several immune functions and also expressed by Treg cells [69]. However, despite the higher expression level of CD73 expressed by maternal cells, they were less prone to inhibit T lymphocyte proliferation, which suggests the involvement of other mediators. Many other factors merit investigation. Among this, the expression of TNF-α-induced gene/protein 6 (TSG-6) has been reported as triggering the M2 skewing [70], the release of exosome [71], or the mitochondrial transfer [72], which should be taken into consideration.

We observed that maternal cells are able to trigger the polarization of the Th22 subset in a stronger manner in comparison to fetal MSC. This population has long been held responsible for triggering inflammatory processes, but more recent articles have indicated the role of Th22 cells in inducing and modifying reparative processes, such as those in the intestinal epithelium [73]. In addition, supernatants from Th22 cells were also shown to enhance wound healing in an in vitro injury model, and this effect was demonstrated to be dependent on IL-22 [74].

We also observed differences between the effects exerted on APC by maternal-derived and fetal-derived cells. More specifically, we reported how both fetal and maternal cells were able to block monocyte differentiation. However, fetal-derived cells were more prone to enhance M2 markers CD163 and CD209, which possibly suggests a different secretion profile between maternal-derived and fetal-derived cells. Prostanoids, and especially PGE2, are among the factors most frequently reported as able to interfere with the Mo-APC differentiation by MSC [49,50] and favoring the acquisition of features that are typical of M2 macrophages such as the increased expression of the M2 marker CD209 [49,75]. In addition, the higher expression of CD200 and Galectin-9 by fetal MSC could account for their enhanced ability to induce an M2-like phenotype due to their immune regulatory action on macrophages [76,77,78].

Lastly, we studied the capacity of maternal and fetal cells to induce an immunogenic response when co-cultured with allogeneic PBMC. hAMSC have been reported to be poorly immunogenic, which is likely due to the absence of co-stimulatory molecules such as CD80 or CD86, and the low level of HLA-DR, that, together, trigger T lymphocyte activation [79,80]. However, previous data reported that low doses of MSC are stimulatory in immunogenicity assays, while higher doses were suppressive [81,82,83]. Both fetal and maternal cells express HLA-ABC and the co-stimulatory molecule CD95, which could confer the low antigen-presenting properties observed. Moreover, it was reported that IFN-γ stimulation augments the expression of HLA-ABC, HLA-DR, and CD40 in hAMSC [84], and the expression of these immunogenic markers could be responsible for their stimulatory activities, as observed for MSC from other sources [85,86].

However, when compared to the T-cell stimulation induced by professional APC such as mDC, induction by both fetal-derived and maternal-derived cells was much lower.

## 5. Conclusions

The present study provides a detailed understanding and comparison of the immunological properties of placental maternal-derived and fetal-derived cells. For the first time, we carried out the comparison using GMP-produced cells, the placental expanded (PLX) cells, specifically designed for clinical application as evidence by the numerous clinical trials in which these cells are used as reported in the ClinicalTrials.gov website, available online: https://clinicaltrials.gov/ (accessed on 04/01/2020) A pitfall of this work could be the reduced number of batches used in this study (one batch of fetal and two batches of maternal GMP products), but this is overcome by the fact that, in this study, we used GMP-prepared cells that are manufactured in a fully controlled 3D system. Each batch is released based on pre-defined release criteria, which allows for consistent and robust cell production. In addition, the use of standardized cell preparations and GMP products with a verified origin (maternal or fetal) is significantly relevant due to the certainty of the origin and standardized isolation and preparation methods. Furthermore, the in vitro efficacy of PLX-R18 was compared with that from 11 preparations of fetal hAMSC, and we observed very similar results, which further strengthens our observations on fetal-derived cells.

There is a huge effort to develop potency assays, which should identify the most “functional” cell for specific applications, including those that evaluate immune features, considering that immunomodulation is a fundamental mechanism of action of MSC [87,88]. Our extensive analysis has highlighted the profound differences in the immunomodulatory properties of MSCs isolated from different sources. We provide evidence that maternal (PLX-PAD) and fetal (PLX-R18) cells had differing immune modulatory properties whereby fetal-derived cells were able to more strongly inhibit T-cell proliferation and cytotoxicity, and induce the switch to M2 macrophages, while maternal-derived cells were more strongly able to induce Treg. Considering these results and the fact that MSC have been suggested to act principally through a paracrine mechanism of action [89], the screening of the secretome for selected cytokines and molecules could represent a feasible potency assay to determine the potential application of cells based on their immune modulatory properties.

Whether or not the immunomodulatory differences observed in vitro could reflect those in vivo remains to be verified. Actually, both PLX-PAD and PLX-R18 are applied in several clinical trials (clinicaltrial.gov), but not for the same pathology. A strong characterization of these cells would be necessary in order to identify which cell typology for which disease. Previously published data by our group reported that hAMSC can exert a strong immune modulatory effect in a different mouse model of autoimmune diseases like encephalomyelitis, colitis, and arthritis [90]. Fetal PLX-R18 cells have many similarities with hAMSC and, thus, these cells could be used in a preclinical model of graft versus host disease (GVHD), while PLX-PAD cells due to the high release of the pro-angiogenic factors vascular endothelial growth factor (VEGF) and Angiopoietin have been tested in the animal model of hind limb ischemia [39,40].

To date, PLX-PAD are used or have been used in clinical trials to treat critical limb-ischemia and hip fracture, with the aim to stimulate angiogenesis to bring oxygenated blood to ischemic tissue, heal damaged muscle, and dampen inflammation, which supports tissue regeneration. Instead, PLX-R18 are used to sustain hematopoiesis to treat incomplete recovery of transplanted hematopoietic cells and acute radiation syndrome. Based on our in vitro results, we could speculate applying the fetal PLX-R18 cells in inflammatory-based diseases, due to their strong immunomodulatory properties. PLX-R18 could dampen the inflammatory environment (M1 macrophages and cytotoxic T cells) and favor inflammation resolution (promoting macrophage polarization to M2) to promote regeneration.

The observed differences underline the importance of the different properties of fetal versus maternal placenta cells, which are fundamental to guide future applications of maternal-derived and fetal-derived cells in different regenerative medicinal approaches.

## Figures and Tables

**Table 1:** Cytokine and chemokine analysis on the effect of either maternal or fetal MSC on PBMC stimulated with CD3CD28 mAbs. Allogeneic PBMC were stimulated with CD3CD28 mAbs for six days and co-cultured or not in the presence of maternal PLX-PAD cells, fetal PLX-R18, or fetal hAMSC cells at a PBMC:MSC ratio of 1:1. After six days, the supernatant was collected and analyzed for the expression of a panel of cytokines and chemokines. Data are indicated as mean ± SD (*n* = 4).

		PBMC CD3CD28	PBMC CD3CD28+ PLX-PAD	PBMC CD3CD28+ PLX-R18	PBMC CD3CD28+ hAMSC
Th1	IFNγ	2521.9 ± 446.2	2021.9 ± 728.2	183.6 ± 91.2	181.2 ± 111.4
	TNFα	1076.0 ± 319.6	168.4 ± 132.2	3.8 ± 0.9	6.1 ± 4.8
Th2	IL-4	1.9 ± 0.5	6.4 ± 2.4	1.9 ± 0.7	1.4 ± 0.4
	IL-5	775.1 ± 456.7	205.6 ± 100.7	35.5 ± 29.4	30.0 ± 20.9
	IL-13	893.6 ± 290.3	992.4 ± 202.6	141.8 ± 79.7	195.9 ± 125.0
Th17	IL-17A	164.9 ± 197.5	60.8 ± 35.3	174.6 ± 111.1	227.6 ± 151.6
Treg	IL-10	115.9 ± 14.6	112.8 ± 26.1	96.9 ± 34.6	117.9 ± 37.5
	TGFβ1	359.4 ± 98.9	931.0 ± 185.3	938.3 ± 61.8	933.7 ± 126.7
Cytotox	GrzB	5778.0 ± 450.6	3490.1 ± 580.6	2397.2 ± 1429.2	2451.2 ± 528.6
	GrzA	1963.5 ± 662.4	1842.7 ± 12.2	1122.5 ± 989.0	640.4 ± 406.5
	RANTES	3108.5 ± 822.7	3937.9 ± 424.5	443.7 ± 143.2	377.2 ± 248.7

## Supplementary Materials

The following are available online at https://www.mdpi.com/2073-4409/9/1/127/s1, Table S1: Cytokine/chemokine profile of maternal and fetal MSCs.
