# Supplementary material for: Mesenchymal Stromal Cells from Fetal and Maternal Placenta Possess Key Similarities and Differences: Potential Implications for Their Applications in Regenerative Medicine
Source: Cells. 2020 Jan 6;9(1):127. doi: 10.3390/cells9010127 (PMC7017205; doi:10.3390/cells9010127)
Supplement: Supplementary file 1 [file cells-09-00127-s001.pdf]

SUPPLEMENTARY TABLE 1

Supplementary Table 1. Cytokine/chemokine profile of maternal and fetal MSCs. PLX-PAD, PLX-R18 and hAMSC cultured for 6 days, experiments were performed in parallel to those performed for Th subset differentiation. The supernatant was collected at day 6 and screened for the expression of cytokine and chemokine using the Flex Set kit by BD Biosciences. Data are represented as mean ± SD (*n* = 4).

	**PLX-PAD**	**PLX-R18**	**hAMSC**
**IFNγ**	2.6±0.6	2.3±1.3	3.3±1.6
**TNFα**	3.3±1.5	2.3±1.7	4.6±3.3
**IL-4**	2.2±0.7	1.4±1.1	2.5±1.5
**IL-5**	0.1±0.1	0.1±0.0	0.1±0.1
**IL-13**	0.2±0.3	0.5±0.5	0.4±0.4
**IL-17A**	28.7±8.2	25.6±12.6	31.7±9.6
**IL-10**	20.6±13.8	2.1±1.1	8.6±4.1
**TGFβ1**	930.0±375.3	1501.3±877.6	1309.9±458.9
**GrzB**	0.1±0.2	0.2±0.4	0.2±0.2
**GrzA**	0.4±0.5	0.4±0.4	0.3±0.3
**RANTES**	14.1±11.6	67.6±35.9	4.5±2.3
